# Exploring the Anthelmintic, Antioxidant, and Cytotoxic Potential of *Khaya grandifoliola* and *Faidherbia albida* Extract Combinations: In Vitro Studies on *Heligmosomoides polygyrus* and *Caenorhabditis elegans*

**DOI:** 10.1155/japr/7208016

**Published:** 2026-01-02

**Authors:** Yamssi Cédric, Noumedem Anangmo Christelle Nadia, Baigomen Christalin, Mounvera Abdel Azizi, Tako Djimefo Alex Kevin, Vincent Khan Payne, Haibo Hu

**Affiliations:** ^1^ Department of Biomedical Sciences, Faculty of Health Sciences, University of Bamenda, Bambili, Cameroon, unibda.net; ^2^ Laboratory of Tropical and Emerging Infectious Diseases, Dschang, Cameroon; ^3^ Department of Microbiology, Haematology and Immunology, Faculty of Medicine and Pharmaceutical Sciences, University of Dschang, Dschang, Cameroon, univ-dschang.org; ^4^ Department of Animal Biology, Faculty of Science, University of Dschang, Dschang, Cameroon, univ-dschang.org; ^5^ Department of Animal Organisms, Faculty of Science, University of Douala, Douala, Cameroon, univ-douala.com; ^6^ Jiangxi Province Key Laboratory of Pharmacology of Traditional Chinese Medicine, National Engineering Research Center for Modernization of Traditional Chinese Medicine - Hakka Medical Resources Branch, School of Pharmacy, Gannan Medical University, Ganzhou, China, gmu.cn

**Keywords:** anthelmintic, *Caenorhabditis elegans*, *Faidherbia albida*, *Heligmosomoides polygyrus*, *Khaya grandifoliola*

## Abstract

**Background:**

Soil‐transmitted helminth infection is endemic in Chad and constitutes a public health problem, particularly among school‐age children. The aim of this study was to evaluate the synergistic anthelmintic activity of the combined extracts of *Khaya grandifoliola* and *Faidherbia albida* used in Chad by traditional practitioners for the treatment of soil‐transmitted helminth infection.

**Methods:**

The anthelmintic tests using combinations of *K. grandifoliola* and *F. albida* followed standard protocol. Combination 1 corresponding to 75% *F. albida* and 25% *K. grandifoliola*, Combination 2 corresponding to 50% *F. albida* and 50% *K. grandifoliola*, and Combination 3 corresponding to 25% *F. albida* and 75% *K. grandifoliola*. The nematocidal activity was assessed on *Heligmosomoides polygyrus* and *Caenorhabditis elegans* using the WMicroTracker. L3 larvae of *H. polygyrus* were obtained after 7 days of coproculture, and L4 of *C. elegans.* One hundred microliter concentrations of extracts, albendazole, and distilled water were brought into contact with 100 *μ*L of *H. polygyrus* suspension in a 96‐well microplate incubated for 20 h at 28°C in the WMicroTracker. The same procedure was adopted for *C. elegans*, but 180 *μ*L of OP50 and 19 *μ*L of *C. elegans* suspension were mixed with 1 *μ*L of extracts and incubated at 20°C in the WMicroTracker. The antioxidant activity was assessed by 2,2‐diphenyl‐1‐picrylhydrazyl (DPPH) radical, iron reducing power (Frap), hydrogen peroxide (H_2_O_2_), and nitric oxide (NO). Cytotoxicity was tested on red blood cells. The phytochemical screening was conducted using both qualitative and quantitative methods. Standard procedures were followed to ensure accuracy and reliability. The analysis was aimed at identifying and measuring the bioactive compounds present.

**Results:**

Combination 2 (50% *F. albida* and 50% *K. grandifoliola*) was the most promising, with an IC_50_ of 0.26 and 0.003 mg/mL, respectively, on *H. polygyrus* and *C. elegans*. This was followed by Combination 3 with an IC_50_ of 0.43 mg/mL for *H. polygyrus.* Combination 2 and Combination 3 with percentage inhibitions of 98.61 ± 0.98 and 99.14 ± 0.60, respectively, for *H. polygyrus* and *C. elegans* did not show a significant difference with respect to albendazole and levamisole. Combination 2 has the capacity to reduce iron with an IC_50_ of 2170 ± 3.73 * μ*g/mL. Ascorbic acid (46.19 ± 0.18 * μ*g/mL) used as a reference molecule shows a greater capacity than that of combination 2. With regard to H_2_O_2_, Combination 2 inhibited H_2_O_2_ slightly less (IC_50_: 0.114 ± 0.02 * μ*g/mL) than ascorbic acid (IC_50_: 0.108 ± 0.03 * μ*g/mL). As for DPPH, the combination had a moderately low scavenging activity (17.64 ± 0.18 * μ*g/mL) compared with ascorbic acid, which had a good scavenging activity (7.23 ± 3.73 * μ*g/mL). Combination 2 had a very weak effect on NO (IC_50_: 103.5 *μ*g/mL) compared with ascorbic acid (54.22 ± 3.74 * μ*g/mL).

**Conclusion:**

This study provides scientific evidence supporting the traditional use of combined extracts from the two plants in treating helminthiasis. However, nematodes differ in structure, composition, and physiology. Further in vivo studies are needed to understand how Combination 2 affects nematode larvae.

## 1. Introduction

Intestinal parasitosis, generally caused by helminths, is a serious health problem [1]. It has been estimated, according to the World Health Organization (WHO), that 3 billion people are infested by intestinal parasites worldwide [2]. Helminthiasis and intestinal parasitosis are mainly caused by geohelminths, namely, *Necator americanus*, *Ancylostoma duodenale*, *Ascaris lumbricoides*, *Trichuris trichiura*, *Schistosoma haematobium*, and *Schistosoma mansoni*, and are found in most tropical and subtropical countries [3]. In Africa, the prevalence of intestinal parasitosis varies. Some studies have shown a prevalence of 40.1% in Burkina Faso, 31.3% in Senegal, and 36.5% in Côte d′Ivoire [4]. Approximately 10 million people suffer from helminthiasis, according to the schistosomiasis and helminthiasis control program (PNLSHI) in Cameroon [5]. In Chad, neglected tropical diseases are still a major public health problem. Helminthiasis continues to be a significant public health problem in Chad, primarily among rural and vulnerable communities [6]. These infections are also a major contributor to morbidity, including anemia, malnutrition, and intellectual impairment, adding to the socioeconomic costs of infected populations in Tchad [7].

A National Coverage Survey in 2017 and 2018 showed that the prevalence of geohelminths varies between 0% and 32.8% [5]. More than 381,000 preschool and school‐age children need treatment according to the mass coverage rate for neglected tropical diseases in 2016 [8]. It is estimated that the overall infestation rate is 35.87% in southern and central Chad, with 16.41% *A. lumbricoides*, 14% *S. mansoni*, and 6.53% *Hymenolepsis nana* being the most commonly found helminths [9]. The Chadian authorities have expanded access to universal health coverage to improve access, quality, and availability of care for the underprivileged. This program, launched in 2011, has been officially open to the most disadvantaged since February 28, 2025. Universal health coverage allows people not to pay directly at the time of care, which is a modern strategy to break down financial barriers and prevent catastrophic health expenditures for the Chadian population [10]. In the face of disease, the body needs an antioxidant defense system to prevent or combat various attacks on the body [11]. Oxidative stress is implicated as a cause or complication of helminthiasis. Oxidative stress results from an imbalance between oxidative systems and the antioxidant capacity of cells. When affected by parasites, the body secretes free radicals in an abusive manner against the parasites. However, these free radicals are toxic for the organism and attack not only DNA, leading to different types of lesions, but also proteins and lipids, altering their function [12].

For years, medicinal plants have been important sources for the search for new effective molecules for the treatment of several diseases [13]. They have an anthelmintic advantage in the treatment of helminthiasis because of their low toxicity and availability. *Khaya grandifoliola* (a large deciduous tree of the family Meliaceae) and *Faidherbia albida* (a leguminous tree belonging to the Fabaceae family) are traditionally used in combination by local populations in central, western, and southern Chad for the treatment of helminthiasis. Studies have shown that the aqueous and organic extracts of *F. albida* and *K. grandifoliola* bark have antimicrobial activity [14]. A plant with multiple pharmacological properties (e.g., antimicrobial, anti‐inflammatory, antioxidant, anthelmintic, and antimalarial) can be used to treat multiple diseases or symptoms. In Cameroon, Guy‐Armand et al. [15] showed that *K. grandifoliola* has antiplasmodial activity. Both *K. grandifoliola* and *F. albida* contain bioactive compounds with potential anthelmintic properties. Several studies have identified bioactive compounds in *K. grandifoliola* that contribute to its medicinal properties, including anthelmintic activity [16, 17]. Flavonoids possess antioxidant and anti‐inflammatory properties that may enhance anthelmintic activity [18]. *F. albida* has also been reported to contain bioactive compounds with anthelmintic effects [19]. Alkaloids have neurotoxic effects on parasites, disrupting their nervous systems [20]. Tannins reduce the viability of parasite eggs and interfere with their development [21].

*Caenorhabditis elegans* and *Heligmosomoides polygyrus* are excellent parasitic nematode models for anthelmintic drug discovery and for elucidating the mechanism of action due to their similarity to parasitic species [22]. The rapid cycle and ease of culture make these nematodes an excellent tool for the anthelmintic activity of substances [23]. The present study was undertaken to evaluate the anthelmintic activity of the combination of *K. grandifoliola* and *F. albida* stem barks in order to determine potential synergistic or antagonistic effects and to provide scientific justification for their traditional use in combination within the Chadian pharmacopeia for the treatment of helminthiasis.

## 2. Materials and Methods

### 2.1. Animal Material

*H. polygyrus* was generously provided to us by Rick Maizels, a professor of parasitology at the Wellcome Centre for Integrative Parasitology within the University of Glasgow′s Institute of Infection, Immunity, and Inflammation. *C. elegans* was provided by the Caenorhabditis Genetics Center (CGC) at the University of Minnesota, in Minneapolis, MN. *H. polygyrus* is a parasitic roundworm that predominantly affects rodents, especially mice (*Mus musculus* and *Apodemus* species). It lives in the small intestine, causing long‐term infections. This parasite is extensively used in research to study host–parasite dynamics, immune responses, and treatments for parasitic infections. A free‐living nematode, *C. elegans* (Bristol N2), is a genetically modified model strain. *Escherichia coli* strain OP50 was used to feed the latter. *H. polygyrus*, a parasite of Swiss strain albino mice (*M. musculus*), was maintained in the laboratory in artificially infested mice.

### 2.2. Preparation of Extract

Using an electric balance (SF‐400), 100 g of *K. grandifoliola* and *F. albida* powder were weighed separately and placed in a 2‐L container. One liter of distilled water boiled at 100°C was introduced into the bucket containing the powder and then hermetically sealed until it cooled. The homogenate was filtered using a sieve and Whatman paper No. 3. The filtrate obtained was placed in an oven at 45°C for drying [24]. Although the traditional use of the combined extract of *K. grandifoliola* and *F. albida* follows a 1:1 (50:50) ratio, three combination ratios (75:25, 50:50, and 25:75) were experimented with in the present study in order to assess synergistic, additive, or antagonistic actions. This is based on the traditional pharmacological practice with the view to optimizing therapeutic effect and maximizing the knowledge of the phytochemical contribution of both plants. These same approaches have been employed by other researchers [25], highlighting the importance of altering component ratios to achieve the most effective formulation. The plant combinations were prepared by weighing the evaporated powders and mixing them as follows: Combination 1 corresponding to 75% *K. grandifoliola* and 25% *F. albida*, Combination 2 corresponding to 50% *K. grandifoliola* and 50% *F. albida*, and Combination 3 corresponding to 25% *K. grandifoliola* and 75% *F. albida*, as shown in Table 1.

**Table 1:** Proportion of the combination.

**Proportions**	**Aqueous extract of *K. grandifoliola***	**Aqueous extract of *F. albida***
Combination 1	75%	25%
Combination 2	50%	50%
Combination 3	25%	75%

### 2.3. *H. polygyrus* L3 Larvae Culture

The coproculture technique used here is that described by Christalin et al. [19]. Five grams of fresh feces were placed in a mortar with an equivalent quantity of charcoal and a few drops of distilled water and crushed. The resultant semisolid mixture was placed on a filter paper in a Petri dish and placed into a box. These boxes were placed in an incubator at 28°C. Seven days later, L3 larvae were obtained.

### 2.4. *C. elegans* Culture

*C. elegans* was grown on solid NGM (Nematode Growth Medium). The NGM was poured onto Petri dishes and inoculated with 200 *μ*L of a fresh culture of *E. coli* OP50. The strains were maintained in an incubator at 20°C [26].

### 2.5. Synchronization of *C. elegans*

Synchronization was performed using the bleaching method [27]. The synchronization of *C. elegans* ensures that all worms in a population are at the same developmental stage, which is essential for experiments that require reliable and consistent results. Gravid nematodes grown on NGM‐Agar medium containing OP50 were washed with S‐basalt solution. Sodium hypochlorite solution and 1 mL of NaOH (5 M) were used for bleaching the worms.

### 2.6. Anthelmintic Activity of Combined Extracts on L3 Larvae of *H. polygyrus* and L4 *C. elegans* Larvae

The anthelmintic tests using combinations of *K. grandifoliola* and *F. albida* were carried out using the combination protocol described in Table 1. The test on L3 and L4 was carried out as described by Santos et al. [28] with some modifications. The concentrations tested were the same as in the original method: 2.5, 1.25, 0.635, 0.312, 0.151, and 0.078 mg/mL for L3 and 100, 50, 25, 12.5, 6.25, and 3.125 *μ*g/mL for L4. However, in this study, the stock solutions used to prepare these concentrations were obtained by first mixing the extracts in different proportions, as shown in Table 1. Combination 1 consisted of 75% *K. grandifoliola* and 25% *F. albida*; Combination 2 consisted of 50% *K. grandifoliola* and 50% *F. albida*; and Combination 3 consisted of 25% *K. grandifoliola* and 75% *F. albida*. Fifty worms were placed in individual wells of a multiwell plate (usually a 96‐well plate) containing 200 *μ*L of the testing solution and incubated for 24 h in a Worm MicroTracker system to monitor the movement and behavior of the worms. This system uses advanced imaging and tracking algorithms to measure parameters like motility, frequency of movement, and other locomotor activities.

The percentage inhibition (PI) of larval motility was determined by the following formula:

PI=Larvae mobility in treated wells−Mobility of larvae in the negative controlMobility of larvae in the negative control×100.

### 2.7. In Vitro Antioxidant Activity of the Most Active Combination

#### 2.7.1. Scavenging Activity of 2,2‐Diphenyl‐1‐Picrylhydrazyl (DPPH) Radicals

The method for measuring antioxidant power using DPPH is based on the compound′s ability to donate hydrogen to the DPPH radical. The presence of these DPPH radicals gives rise to a dark violet coloration of the solution, which absorbs around 517 nm. The change in color from violet to yellow is proportional to the antioxidant power [29]. The antioxidant activity related to the scavenging effect of the DPPH ˙ radical is expressed in PI using the following formula:

PI=A01−AAO×100,

where *A*0 is the average absorbance in negative control wells and *A*1 is the absorbance in wells containing extracts/vitamin C.

#### 2.7.2. FRAP Test (Iron Power Reduction Test)

The FRAP method is based on the chemical reduction of Fe(III) ions present in the potassium ferricyanide complex K3Fe(CN)6 to Fe(II) ions using the method described by Chung et al. [30]. The absorbance of the reaction medium was determined at 700 nm. An increase in absorbance corresponds to an increase in the reducing power of the extracts tested.

#### 2.7.3. Nitric Oxide (NO) Radical Inhibition Test

NO plays a key role in the inflammatory response by activating several signaling pathways that exacerbate the deleterious effects of oxidative stress. Nitrite (NO_2_^−^) accumulation is considered to be an indicator of NO production in the culture medium. Unstable physiological NO is rapidly oxidized to NO_2_^−^ and nitrate (NO_3_^−^). The latter is then converted to NO_2_^−^ by the action of NO_3_^−^reductase [31].

#### 2.7.4. Hydrogen Peroxide (H_2_O_2_)

One of the most common methods for assessing H_2_O_2_ scavenging capacity is based on the absorption of this molecule in the UV range [32]. H_2_O_2_ is a nonradical oxygen derivative and is considered toxic for cells because it allows the formation of hydroxyl radicals (HO•) inside the cell.

#### 2.7.5. Cytotoxicity Test for the Most Active Combination

The hemolysis test on erythrocytes was carried out according to the method described by Sinha et al. [12]. Briefly, 500 *μ*L of the suspension of healthy erythrocytes from fresh blood collected from rats prepared at 4% hemocrit was added in the presence of 500 *μ*L of extracts at different concentrations (from 31.62 to 1000 mg/mL) in hemolysis tubes. Under the same conditions, 10% Tween (for 100% hemolysis) was used as a positive control. The mixture was incubated at 37°C for 3 h. After incubation followed by centrifugation at 2500 rpm for 3 min, the supernatant of the solution was introduced into the microplates. The absorbance of the supernatant corresponding to hemoglobin release was read at 540 nm using a microplate reader (Biobase). The hemolysis rate was calculated using the following formula.

%hemolyse=OD extract−OD negative controlOD negative control×100,

where OD is the optical density.

### 2.8. Phytochemical Screening of the Most Active Combination

Quantitative and qualitative phytochemical screening was done according to the method described by Shaikh and Patil [33]. The qualitative analysis used standard chemical assays to identify the presence of major categories of secondary metabolites, including alkaloids, flavonoids, tannins, saponins, glycosides, phenols, terpenoids, and steroids. These assays depended on specific color changes or the formation of precipitates upon introducing certain reagents. In the quantitative analysis, spectrophotometric methods were employed to measure the concentrations of essential phytochemical groups such as total phenols, flavonoids, and tannins. All procedures were conducted in triplicate to guarantee the accuracy and reproducibility of the results.

### 2.9. Ethical Consideration

All authors hereby declare that the “Principles for the Care of Laboratory Animals” (NIH Publication No. 85‐23, Revised 1985) have been followed, as well as specific national laws, where applicable. All experiments were reviewed and approved by the Institutional Ethics Committee for Research on Human Health Review Board (N°4840/CEI‐UDo/04/2024/T) of the University of Douala.

### 2.10. Statistical Analysis

Microsoft Excel was used to determine the PI based on the activity values. The concentration–response curves generated by graphing the logarithm of the concentration as a function of the percentage inhibition using the GraphPad Prism Version 8 software were then used to calculate the half‐maximum inhibitory concentrations (IC_50_).

## 3. Result

### 3.1. Anthelmintic Activity of Combined Extracts

Table 2 shows the IC_50s_ and PI of motility of L3 and L4 larvae of *H. polygyrus* L3 and *C. elegans* L4 larvae. Combination 2 with a proportion of 50%/50% is the most promising, with an IC_50_ of 0.26 and 0.05 mg/mL, respectively, on *H. polygyrus* L3 and *C. elegans* L4 larvae. This was followed by Combination 3 with an IC_50_ of 0.55 mg/mL for *H. polygyrus* L3 larvae. Combination 2 and Combination 3 with PIs of 98.61 ± 0.98 and 99.14 ± 0.60, respectively, for *H. polygyrus* L3 and *C. elegans* L4 did not show a significant difference to albendazole and levamisole (100 ± 0.0).

**Table 2:** Percentage inhibition and IC_50_ of motility in L3 and L4 larvae of *H. polygyrus* and *C. elegans.*

**L4 *C. elegans***	**Extracts**	**Concentrations (*μ*g/mL)**						**IC**_**50**_	**Levamisole**	**Negative control (water)**
		**3.125**	**6.25**	**12.5**	**25**	**50**	**100**			

Combination	Combination 1	4.23 ± 0.04^a^	8.43 ± 0.08^a^	18.75 ± 1.70^b^	54.33 ± 0.85^c^	89.49 ± 2.08^e^	97.92 ± 2.08^f^	0.83	100	0
	Combination 2	16.45 ± 0.10^a^	35.41 ± 0.85^b^	56.64 ± 0.90^c^	70.83 ± 0.49^d^	90.97 ± 0.98^e^	98.6 ± 0.98^f^	0.26	100^f^	0
	Combination 3	13.77 ± 0.45^a^	15.24 ± 0.50^a^	39.58 ± 0.58^b^	81.82 ± 10.5^c^	84.42 ± 0.17^d^	92.93 ± 2.56^d^	2.60	100	0

**L3 *H. polygyrus***	**Extracts**	**Concentrations (mg/mL)**						**IC**_**50**_	**Albendazole**	**Negative control (S-basalt)**
		**0.078**	**0.156**	**0.312**	**0.625**	**1.25**	**2.5**			

Combinations	Combination 1	12.12 ± 0.06^a^	28.20 ± 0.04^b^	66.67 ± 0.05^c^	78.30 ± 1.57^d^	89.70 ± 0.06^e^	96.30 ± 1.58^f^	0.13	100^f^	0
	Combination 2	28.45 ± 0.14^a^	66.6 ± 0.02^b^	78.30 ± 1.57^b^	89.8 ± 0.04^c^	96.10 ± 0.6^d^	99.10 ± 0.0^d^	0.05	100^d^	0
	Combination 3	15.31 ± 0.07^a^	64.30 ± 0.11^b^	69.9 ± 1.15^b^	76.12 ± 0.03^c^	82.14 ± 0.08^c^	91.14 ± 0.60^d^	0.55	100	0

*Note:* The result is presented as *m*ean ± *s*tandard *d*eviation. Values in the same column with the same superscript letter (a, b, c, d, e, and f) are not significantly different from each other, while those with different letters are significantly different (*p* < 0.05).

### 3.2. In Vitro Antioxidant Potential of Combination 2

Table 3 shows the antioxidant activity of Combination 2. The combination of *K. grandifoliola* and *F. albida* has the capacity to reduce iron with an IC_50_ of 2170 ± 3.73 * μ*g/mL. Ascorbic acid (46.19 ± 0.18 * μ*g/mL) used as a reference molecule shows a greater capacity than that of Combination 2. With regard to H_2_O_2_, Combination 2 inhibited H_2_O_2_ slightly less (IC_50_: 0.114 ± 0.02 * μ*g/mL) than ascorbic acid (IC_50_: 0.108 ± 0.03 * μ*g/mL). As for DPPH, the combination had a moderately low scavenging activity (17.64 ± 0.18 * μ*g/mL) compared with ascorbic acid, which had a good scavenging activity (7.23 ± 3.73 * μ*g/mL). Finally, on NO, Combination 2 had a very weak effect (IC_50_: 103.5 *μ*g/mL) compared with ascorbic acid (54.22 ± 3.74 * μ*g/mL).

**Table 3:** Antioxidant activity of Combination 2.

**Treatment**	**DPPH**	**FRAP**	**Peroxide (H**_**2**_ **O**_**2**_ **)**	**NO**
IC_50_ (*μ*g/mL)				
Vitamin C	7.23 ± 3.73	46.19 ± 0.18	0.10	54.22 ± 6.18
Combination 2	17.64 ± 0.18	2170 ± 34.03	0.11	103.5

### 3.3. Cytotoxicity of Combination 2 on Red Blood Cells of Combination 2

**Figure 1:**
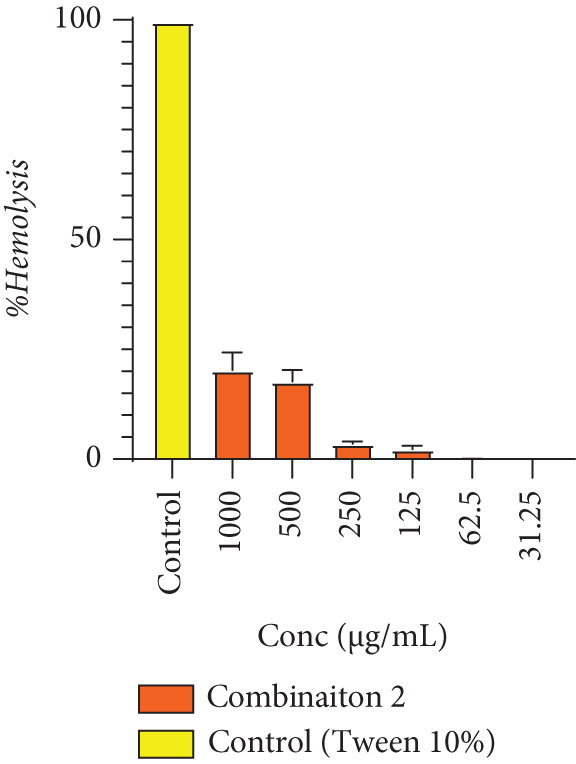
Percentage of hemolysis of red blood cells in Combination 2.

Figure 1 shows the percentages of hemolysis of red blood cells in Combination 2, which was the one presenting the best anthelminthic activity. It can be seen from this figure that hemolysis varies slightly with increasing concentration and is much lower than with Tween (10%), which is the positive control that strongly promotes hemolysis. Combination 2 does not induce red blood cell hemolysis at concentrations of 62.5 and 31.25 *μ*g/mL.

### 3.4. Qualitative Phytochemical Content of Combination 2

Table 4 shows the qualitative results of the phytochemical test for Combination 2. This table shows that Combination 2 does not have quinones but does have all the polyphenolic compounds.

**Table 4:** Qualitative phytochemicals of Combination 2.

**Metabolites**	**Combination 2**
Alkaloids	+
Phenols	+
Tannin	+
Quinones	−
Saponins	+
Anthryamines	+
Flavonoids	+

### 3.5. Quantitative Phytochemical Testing of Combination 2

**Figure 2:**
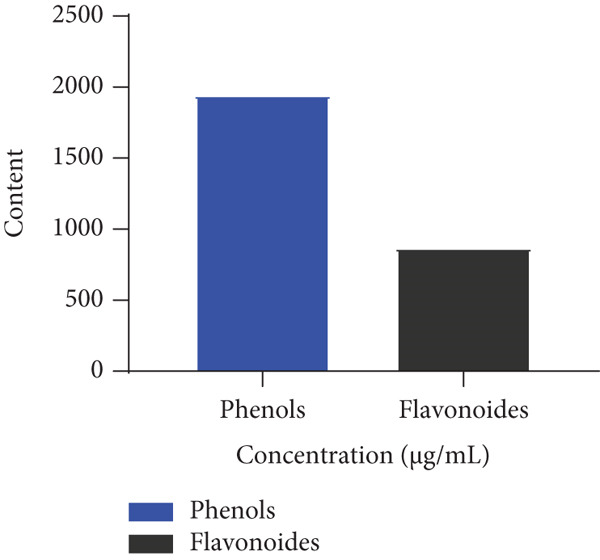
Determination of total phenols and flavonoids in Combination 2.

Figure 2 shows the determination of total phenols and flavonoids in Combination 2. It can be seen from this figure that Combination 2 of the two plants contains a high quantity of phenols and flavonoids with an average level of 1942 ± 72.80 and 867.1 ± 19.47, respectively.

## 4. Discussion

Stem bark′s traditional use in Chad and its abundance in secondary metabolites including phenols, flavonoids, tannins, and alkaloids all of which have been connected to anthelmintic and antioxidant properties justify its selection. Bark is a valuable source of bioactive chemicals because it frequently accumulates these substances as a natural defense against stress and infections. To guarantee effectiveness and sustainability, future research should also evaluate other plant elements, such as leaves or seeds, as collecting bark may jeopardize plant survival. According to earlier research, *K. grandifoliola* includes a variety of phytochemicals, including flavonoids, which have anthelmintic and antioxidant qualities [16, 19]. Flavonoids impair the metabolism of parasites and aid in antioxidant defense. Similarly, it has been discovered that *F. albida* contains phenolic chemicals that have direct anthelmintic actions [19]. In helminths, alkaloids may affect neuromuscular function. These chemicals′ existence in both plants supports their usage in traditional medicine and offers a logical explanation for the nematocidal effect that was reported in this investigation. Their traditional co‐use in Chad is justified by evaluating their efficacy alone and in combination, which shows the potential for synergistic interactions where the combined extracts show higher activity than each plant alone.

Among the tested combinations, Combination 2 (50% *F. albida* and 50% *K. grandifoliola*) demonstrated the most potent anthelmintic activity, with IC_50_ values of 0.26 mg/mL for *H. polygyrus* and 0.003 mg/mL for *C. elegans*. These results are noteworthy, as the activity of this combination was comparable to standard anthelmintics such as albendazole and levamisole. This level of activity is comparable to that of standard anthelmintics such as albendazole, which typically shows IC_50_ values ranging between 0.01 and 0.5 mg/mL depending on the parasite and assay conditions [34]. The significant reduction in larval motility and survival suggests a synergistic or additive effect between the phytochemicals present in the two plants, validating their traditional combined use. In a study by Christalin et al. [19] who reported the anthelmintic activity of extracts of *Khaya anthotheca* and *F. albida* used in Chad by traditional healers for the treatment of helminthiasis from the same genus as *K. grandifoliola*, it was demonstrated that the aqueous extracts of *K. anthotheca* and *F. albida* on *C. elegans* showed IC_50_ values of 0.2775 and 0.5115 mg/mL, respectively, which are significantly higher (i.e., less potent) than the 0.003 mg/mL observed in our Combination 2, indicating enhanced efficacy in the combined preparation.

The observations not only provide an excellent basis for investigations to demonstrate such activity in vivo but also strongly support the idea of Subedi [35] that *K. senegalensis* has considerable potential as a source of anthelmintic medicinal therapies for livestock. The observed differences in activity between combinations further support the hypothesis that the ratio of plant extracts influences bioefficacy. Combination 3 (75% *K. grandifoliola* and 25% *F. albida*) also showed strong activity, albeit slightly lower than Combination 2. This trend implies that *K. grandifoliola* may contribute significantly to the anthelmintic effect, but the inclusion of *F. albida* enhances the activity possibly through complementary or synergistic mechanisms. The effectiveness against both parasitic (*H. polygyrus*) and free‐living (*C. elegans*) nematodes also highlights the potential broad‐spectrum activity of this plant mixture [36]. This study suggested that the compounds could work by disrupting the integrity of the worm′s cuticle, thus leading to parasite mortality. The anthelmintic action was attributed to the plant′s alkaloids and saponins, which have been shown to interfere with parasite metabolism, causing immobilization and death.

The antioxidant assays revealed that Combination 2 also exhibited moderate antioxidant properties, particularly in the H_2_O_2_ scavenging (IC_50_ = 0.114 * μ*g/mL) and iron‐reducing (IC_50_ = 2170 * μ*g/mL) tests, although these were less potent than the reference compound, ascorbic acid. These findings are relevant because oxidative stress plays a role in host–pathogen interactions and in the pathology of helminth infections. Thus, the antioxidant activity of the combination may offer additional therapeutic benefits by modulating inflammation and tissue damage during infection. Antioxidants are essential in fighting helminth infections by alleviating oxidative stress in both the host and the parasite. They protect the host′s tissues from damage due to parasitic infestations. Antioxidants can also impair parasite defenses, increasing their susceptibility to immune reactions and treatment. Furthermore, they may interfere with the parasite′s metabolism, helping to prevent its growth and reproduction. Once in the human body, helminths produce free radicals that are not only toxic to themselves but also to the body. Hence, the need to assess the antioxidant activity of Combination 2, which was found to be the most active. The PI of the free radical for Combination 2 was lower than that of the standard for all the concentrations used. Combination 2 showed good scavenging activity (IC_50_: 17.64 *μ*g/mL) but less than ascorbic acid (IC_50_: 7.23 *μ*g/mL). This is justified by the combination of the two extracts from the two plants, which showed an increase in polyphenolic compounds. It was demonstrated by Bougandoura and Bendimerad [37] that antioxidant molecules such as ascorbic acid and other molecules reduce and decolorize DPPH due to their ability to give up hydrogen. The molecules contained in Combination 2 are probably responsible for the antioxidant activity. In the DPPH and NO scavenging assays, the combination of extracts showed less capacity to scavenge free radicals than ascorbic acid did. Similar results were obtained by Guy‐Armand et al. [15] who reported that the ethanol and aqueous extracts of the stem bark of *K. grandifoliola* were less effective than ascorbic acid at scavenging free radicals. Our results, however, contradict those of Kodjio et al. [38], who showed that ascorbic acid had a more potent DPPH scavenging capacity. The disparity could be explained by the low content of phenolic compounds and flavonoids, which can release H+ to aid in the scavenging of DPPH. These observations corroborate those obtained by Guy‐Armand et al. [15], where *K. grandifoliola* extracts showed lower scavenging activity than ascorbic acid. Bougandoura and Bendimerad [37] also demonstrated that the reducing power of a compound can serve as a significant indicator of its potential antioxidant activity. Furthermore, the reduction of Fe^3+^ could also be due to the increase in absorbance. The greater the absorbance, the greater the reduction, demonstrating that an increase in absorbance indicates an increase in the reducing power tested. NO is an antioxidant indicator. Combination 2 (IC_50_: 143 *μ*g/mL) also showed a lower inhibitory capacity than ascorbic acid. This result could be justified by a combination of polyphenolic compounds contained in the two plants, which increased the reducing power of NO. With regard to iron‐reducing power, Combination 2 IC_50_ was 2170 ± 34.03 * μ*g/mL compared with ascorbic acid 46.19 ± 0.18 * μ*g/mL.

H_2_O_2_ is a chemical compound with the formula H_2_O_2_, a neutralizing source of harmful hydroxyl free radicals. Combination 2 showed an inhibitory effect comparable to that of vitamin C (with an IC_50_ of 2110 ± 1.89). This means that these extracts have little power to inhibit H_2_O_2_. These observations do not corroborate those obtained by Guy‐Armand et al. [15], where the extract of *K. grandifoliola* showed a higher scavenging activity than ascorbic acid. This could be justified by climatic and soil edaphic factors, as the same plant is harvested in different environments. Bougandoura and Bendimerad [37] demonstrated that the fact that a compound has a reducing power is a significant indication of its potential antioxidant activity. This is advantageous to the host organism since, despite its involvement in the body′s defense, H_2_O_2_ is frequently poisonous.

Cytotoxicity tests are crucial for assessing the safety and efficacy of compounds or medicinal plants in combating helminth infections. They help determine if the compounds can specifically target and destroy parasitic cells while sparing host cells. These tests ensure that potential treatments are safe for the host. Moreover, they aid in identifying effective compounds with minimal harmful effects on the host organism. Extracts with anthelmintic activity may be toxic to the organism, this is why cytotoxicity was assessed. The use of a drug is justified not only by its effectiveness in treating the disease but also by its harmlessness to the body. To verify the safety of Combination 2, the cytotoxicity test was performed in vitro on erythrocytes. The cytotoxicity test on erythrocytes showed mild hemolytic activity on red blood cells. These results are in agreement with those obtained by Guy‐Armand et al. [15], where *K. grandifoliola* extract showed negligible lysis of erythrocytes. The chemical composition of Combination 2 revealed the presence of flavonoids, phenols, tannins, saponins, and alkaloids. However, quinones were absent from extracts of both plants. Numerous studies have revealed the importance of polyphenols, particularly tannins, in pest control [39, 40]. Studies have shown that carotenoids, triterpenes, saponins, steroids, coumarins, tannins, and other chemical compounds in plants, such as glycosides, are essential elements of public health. Overall, Combination 2 presents variable polyphenolic compounds. Polyphenolic parameters are secondary metabolites that act by preventing or controlling the mechanism involved in oxidative stress causing multiple disorders in the host organism [41]. Phenolic compounds and flavonoids were meticulously examined in this investigation due to their status as prominent phytochemical categories that possess well‐documented anthelmintic and antioxidant attributes. It is established that phenolic compounds can disrupt the metabolic processes of parasites by interacting with proteins, inhibiting enzymatic functions, and compromising structural elements such as cuticles and egg membranes. Conversely, flavonoids not only manifest direct antiparasitic activities but also serve as potent antioxidants, mitigating the oxidative stress frequently associated with helminthic infections. Combination 2 showed an increase in phenol and flavonoid levels. This could be explained by the soil constituents and seasonal conditions in Chad. This justifies the need to combine the two plants. Polyphenolic parameters are secondary metabolites that act by preventing or controlling the mechanism involved in oxidative stress causing multiple disorders in the host organism [41].

## 5. Conclusion

This study provides scientific support for the traditional Chadian use of *K. grandifoliola* and *F. albida* stem barks against helminthiasis, showing that the 1:1 combination had the strongest anthelmintic effect, comparable to standard drugs, while also demonstrating antioxidant potential. These findings suggest that such plant combinations could serve as promising complementary treatments for soil‐transmitted helminth infections, especially in resource‐limited settings. However, further in vivo studies are needed to confirm their safety, efficacy, and mechanisms of action before clinical application.

## Disclosure

All authors read and approved the final manuscript.

## Conflicts of Interest

The authors declare no conflicts of interest.

## Author Contributions

Y.C. conceived the idea, designed the experiments, performed the experiments, analyzed and interpreted the data, drafted the manuscript. N.A.C.N. conceived the idea, designed the experiments, drafted the manuscript. T.D.A.K. performed the experiments, drafted the manuscript. V.K.P. conceived the idea, designed the experiments. H.H. conceived the idea, designed the experiments, analyzed and interpreted the data, drafted the manuscript. B.C. and M.A.A. performed the experiments.

## Funding

This study was partially funded by the Key University–Local Cooperation Project of Science and Technology Plan Project of Ganzhou (2025XDCE0005) and Jiangxi Provincial Natural Science Foundation (Grant No. 20252BAC240465).

## Data Availability

Data is available upon request from the authors.
